# The Relation between Hæmic and Organic Cardiac Murmurs

**Published:** 1896-01-04

**Authors:** Solomon C. Smith

**Affiliations:** Physician to the Westminster General Dispensary.


### The Relation Between Hæmic and Organic Cardiac Murmurs.

By Solomon C. Smith, M.D., M.R.C.P., Physician to the Westminster General Dispensary.

In considering this matter it is well to bear in mind at the outset that, in a sense, all cardiac bruits are hæmic. Broadly, there are two ways in which sounds are produced by the flow of fluids through tubes or into cavities, (1) by the vibration of a free edge or membrane, or of some projecting substance floating in the stream, in which case the vibration is primarily conducted into the base to which this membrane or edge is attached, and only secondarily conveyed to the blood stream; and (2) by the flow of a comparatively narrow stream of fluid into a wider cavity, producing what has been termed a fluid vein. In this case the primary vibration is in the fluid, and passes in the direction of its flow, and only secondarily affects the walls of the vessels and cavities in which the fluid lies.

The facility with which sounds are produced in this last way no doubt greatly depends on the character of the circulating fluid. We know as a fact that in some conditions of blood the slightest pressure of the stethoscope, either upon an artery or a vein, suffices to set up a roaring bruit, while in other states it is with great difficulty that anything of the sort can be produced, and we may fairly believe that the diminished density and viscosity of the blood, which occur in certain forms of chlorosis and anæmia, greatly favour the development of such bruits, although their production also depends in part upon the condition of the vessels in regard to tension.

In any case, it is clear that one large factor in the mechanism by which these two classes of bruits—the organic and the functional—are produced is the same, and that their differentiation is often a matter of probability and knowledge of what is likely to occur, rather than of any certain characteristic in the sound itself. If it were true that regurgitation through a valve never took place except in presence of organic change in the heart, one's task would be simplified; but it is well known that in many cases a temporary dilatation of the ventricle may cause an incompetence of the auriculo-ventricular valves, the resulting sound being precisely similar in its method of production to what takes place when the valves are rendered incompetent by organic puckering. There is, however, this difference, that while in organic disease there is usually a certain amount of hypertrophy, and the apex is firmly held against the chest walls during the production of the bruit, so that this is conducted far to the left along the ribs, in functional maladies such is not usually the case.

It must then be remembered that a regurgitant bruit is always in reality organic resulting from a leaky valve, and thus in regard to it the problem does not lie, as it is so often spoken of as doing, between hæmic and organic, but between recoverable and unrecoverable valvular incompetence.

In stenosis the condition of affairs is different. Whatever be the anatomical condition by which a narrowing of the orifice is produced, the mode in which the resulting bruit arises is the same, viz., by the production of twirls and eddies in the blood stream, some portions of the blood being projected with greater rapidity through the more slowly moving mass, in the form of fluid veins, by which means sonorous vibrations are set up. This, however, is precisely the manner in which the true hæmic bruits of anæmia are caused, hence the difficulty of pronouncing with certainty as to the origin of a sound by means of its character alone.

It is to be noted that a bruit produced in the blood stream, as most direct bruits are, tends to be conducted in the direction of the current, and that while such a murmur may be very soft it may, on the other hand, be loud and roaring, and accompanied by a thrill, as is well exemplified in the *bruits de diable* so easily elicited in the neck in cases of chlorosis. When, however, murmurs are produced primarily in the valves, they tend to be conducted to the surface by any dense fibrous tissue in the neighbourhood. There is much reason to believe that the murmur so characteristic of mitral stenosis, viz., the presystolic, is a truly hæmic sound, while some of the other bruits, the early diastolic for example, which have been found associated with that condition may be of a valvular type.

Although an exclusively anæmic bruit is but rarely produced in the aorta, many direct aortic murmurs are examples of the hæmic type of sounds; they are produced by eddies and flow on with the current, while regurgitant murmurs, whether at the aortic or mitral orifice, being produced chiefly by vibration set up in the free edges of the valves, are transmitted to the surface by fibrous tissues to the sternum in the one case and the apex in the other, rather than by the blood stream, although in the case of regurgitant mitral such eddies are also produced in the auricle as to cause a sound which is heard in the back. It is a matter of no small interest to bear in mind that the bruit set up by a valvular lesion may be composed of at least two sounds of different character, into which they may in some cases be analysed by the conduction of each in different directions. The point, then, to be borne in mind is, that in the production of cardiac bruits there are two main factors—vibration of a valve or its remains, or of outgrowths upon it, and vibration in the flowing column of blood—that although these two are probably both engaged in every case, they enter in very different proportions into the production of different morbid sounds, and that so far as an organic bruit depends on the vibration produced in the column of blood, it is to all intents and purposes a hæmic murmur. The distinction, then, between "organic" and "inorganic" must be drawn, not from anything in the sounds themselves, but from the known readiness with which functional bruits are produced at certain localities and at certain points in the heart cycle, from the presence or absence of clearly hæmic bruits in other localities, showing whether or no the blood is in such a condition as to lend itself readily to the production of such sounds, and from the presence or absence of such arterial conditions—such as high tension—as would make the formation of fluid veins difficult or improbable; and from the co-existence of signs of circulatory failure or of such heart conditions as would indicate the presence of the compensatory changes which usually follow organic disease.

